# A new chronology for tsunami deposits prior to the 1700 CE Cascadia earthquake from Vancouver Island, Canada

**DOI:** 10.1038/s41598-022-16842-8

**Published:** 2022-07-22

**Authors:** Koichiro Tanigawa, Yuki Sawai, Peter Bobrowsky, David Huntley, James Goff, Tetsuya Shinozaki, Kazumi Ito

**Affiliations:** 1 Geological Survey of Japan, National Institute of Advanced Industrial Science and Technology (AIST), Site C7, 1-1-1 Higashi, Tsukuba, Ibaraki 305-8567 Japan; 2 Geological Survey of Canada, Natural Resources Canada, Sidney, BC V8L 4B2 Canada; 3 Geological Survey of Canada, Natural Resources Canada, Vancouver, BC V6B 5J3 Canada; 4 School Biological, Earth and Environmental Sciences, University of New South Wales, Sydney, NSW 2052 Australia; 5 School of Ocean and Earth Science, University of Southampton, Southampton, UK

**Keywords:** Environmental sciences, Natural hazards, Solid Earth sciences

## Abstract

Coastal deposits at Tofino, Ucluelet, and Port Alberni in Vancouver Island along the Cascadia subduction zone were re-examined to improve the earthquake history of the southwest coast of Canada. We found sand sheets interbedded within peat and mud, suggesting deposition by strong flows in a low-energy environment. Based on limiting maximum and minimum ages derived from plant macrofossils, the age of one of the sand sheets below the tsunami deposits of the great Cascadia earthquake in 1700 CE was estimated to be 1330–1430 CE. Onshore paleoseismic evidence has been documented in Vancouver Island, northern Washington, and northern Oregon during this period. However, the newly constrained age is between those of coseismic subsidence Y and W events in southern Washington, which have been recognized as the 1700 CE and the penultimate Cascadia earthquakes, respectively. Moreover, the new age partly overlaps with the age of offshore paleoseismic evidence for T2, interpreted to have originated from the penultimate Cascadia earthquake, based on offshore turbidite records. The new chronology prior to the 1700 CE Cascadia tsunami deposit from Vancouver Island contributes to a better understand of the timing of the penultimate Cascadia earthquake.

## Introduction

Geological evidence for megathrust earthquakes and associated tsunamis have contributed significantly to our understanding of the timing, recurrence intervals, rupture areas, and magnitudes of past events associated with subduction zones around the world^1–8^. Numerous studies of paleoseismic evidence such as coseismic subsidence stratigraphy^9–12^, tsunami deposits^13–15^, and turbidites^16–18^ along the Cascadia subduction zone (CSZ) exist. A history of Cascadia plate-boundary earthquakes over the last 10,000 years has been reconstructed based on the ages of paleoseismic evidence mainly from the Pacific coast of the northern United States^5,18–20^. The most recent Cascadia earthquake on 26 January 1700 CE is the best documented of all of these events. Its date was constrained by tree-ring dating^21,22^ in southern Washington and from written records in Japan^23^. Earthquake rupture models have been improved based on tsunami descriptions in Japan^24^, distribution and amount of coseismic subsidence inferred from microfossil analyses^25^, and distribution of tsunami deposits^26^. In contrast, the timing and details of the penultimate CSZ earthquake are less certain^18,27^. Evidence for coseismic subsidence at coastal marshes in southern Washington indicated that the predecessor (event W) of the 1700 CE earthquake (event Y) occurred in 1030–1160 CE^9,28^. However, based on the offshore turbidite chronology, the age of the predecessor (turbidite T2) has been estimated to be 1402–1502 CE^18^. Resolution of this discrepancy in chronology between onshore and offshore evidence is essential for long-term earthquake prediction.

**Figure 1:**
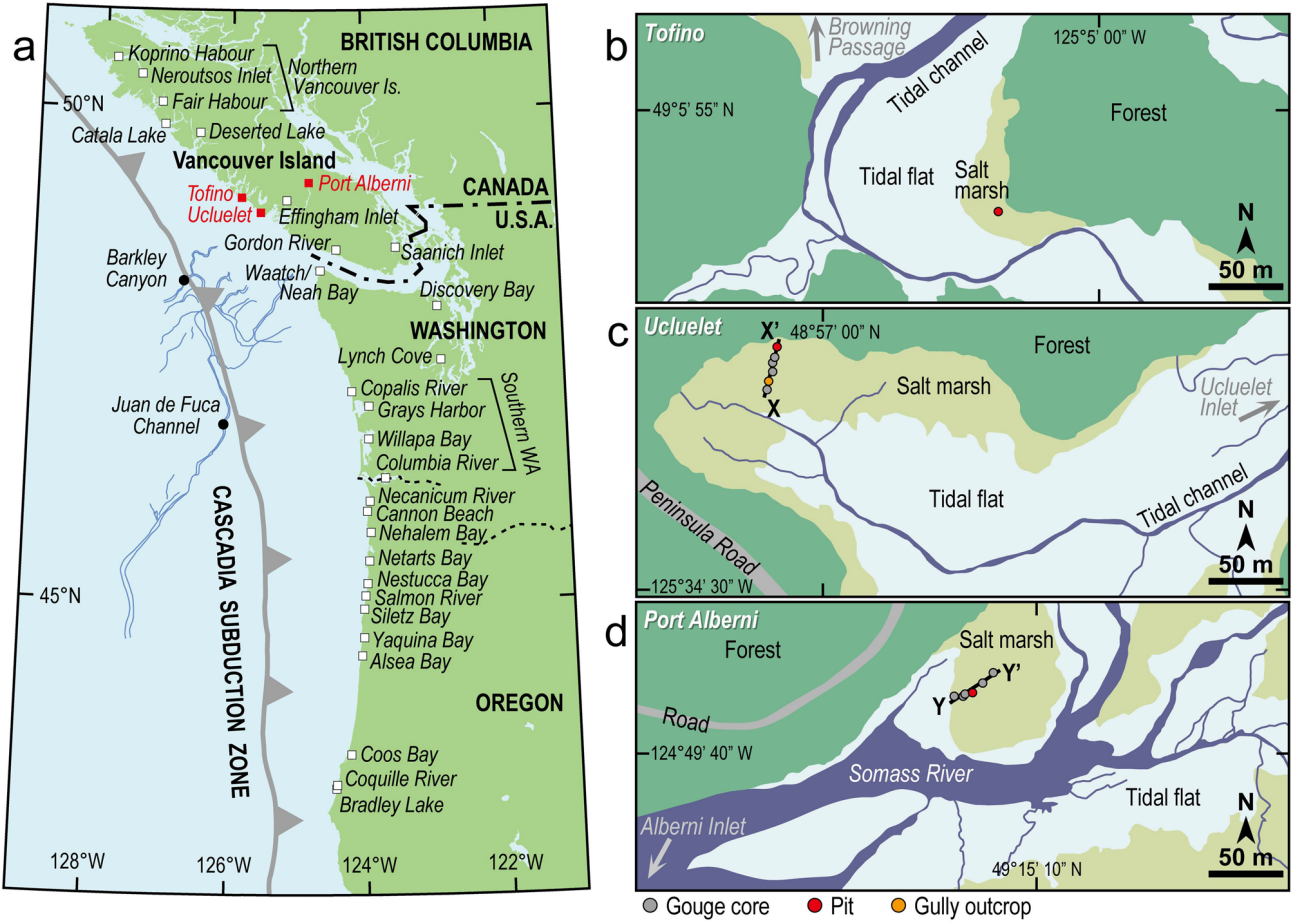
Location map of the study area. (**a**) Map of the Pacific coast of British Columbia, Canada; Washington, and Oregon, USA showing the Cascadia subduction zone. Three red squares show the study sites discussed in this paper. White squares indicate onshore paleoseismic study sites, including coastal lakes and fjord inlets. Solid circles indicate offshore paleoseismic study sites facing southern Vancouver Island and northern Washington. Blue lines indicate submarine canyons and channels facing the southwestern coast of Vancouver Island and northern Washington. The map was generated using Generic Mapping Tools (GMT) version 6^73^. Details of study areas: (**b**) Tofino, (**c**) Ucluelet, and (**d**) Port Alberni. Locations of gouge cores, pits, and an outcrop are indicated by gray, red, and orange circles, respectively.

Paleoseismic evidence has also been documented in coastal marshes, lakes, and fjord inlets along the southwest coast of Canada, aligned with the northern CSZ^27,29–31^. This evidence suggests that the 1700 CE Cascadia earthquake and tsunami affected much of the west coast of Vancouver Island^32–35^. Pioneering studies in 1990s^13,32,36^ reported tsunami deposits associated with the 1964 Alaska and the 1700 CE Cascadia earthquakes from Tofino, Ucluelet, and Port Alberni on Vancouver Island (Fig. 1a). Sand sheets, inferred to be tsunami deposits, have also been documented below the 1700 CE tsunami layers at these sites. However, their depositional ages were not well constrained because of the lack of radiocarbon ages, and the low resolution of radiocarbon dating at the time of analysis. Since the late 1990s, there have been few new paleoseismic studies in this area^27,29–31^ in contrast to the Pacific northwest coast of the United States. New paleoseismological studies are therefore needed to improve the history of earthquakes along the southwest coast of Canada.

In this study, we aim to provide more precise ages for event deposits that may be attributed to the penultimate Cascadia earthquake. In 2015 and 2016 we re-examined coastal deposits within salt marshes at Tofino, Ucluelet, and Port Alberni (Fig. 1b–d).

### Sedimentological and chronological analyses, and the correlation with previous studies

Deposits beneath the salt marshes in the three study sites are characterized by sandy clay with overlying peat and mud. Sand sheets are interbedded within the peat and mud, which suggests that they have been deposited by strong flows including tsunamis, storm surges, and floods in these low-energy environments. Two visible sand sheets were observed in Tofino, four in Ucluelet, and three in Port Alberni. We constrained the depositional ages of sand sheets using a combination of ^137^Cs analysis and ^14^C dating of plant macrofossils and insects. We studied the correlation with previous studies in 1990s^13,32^ that reported tsunami deposits associated with the 1964 Alaska, the 1700 CE Cascadia, and older earthquakes from nearby sites.

#### Tofino

**Figure 2:**
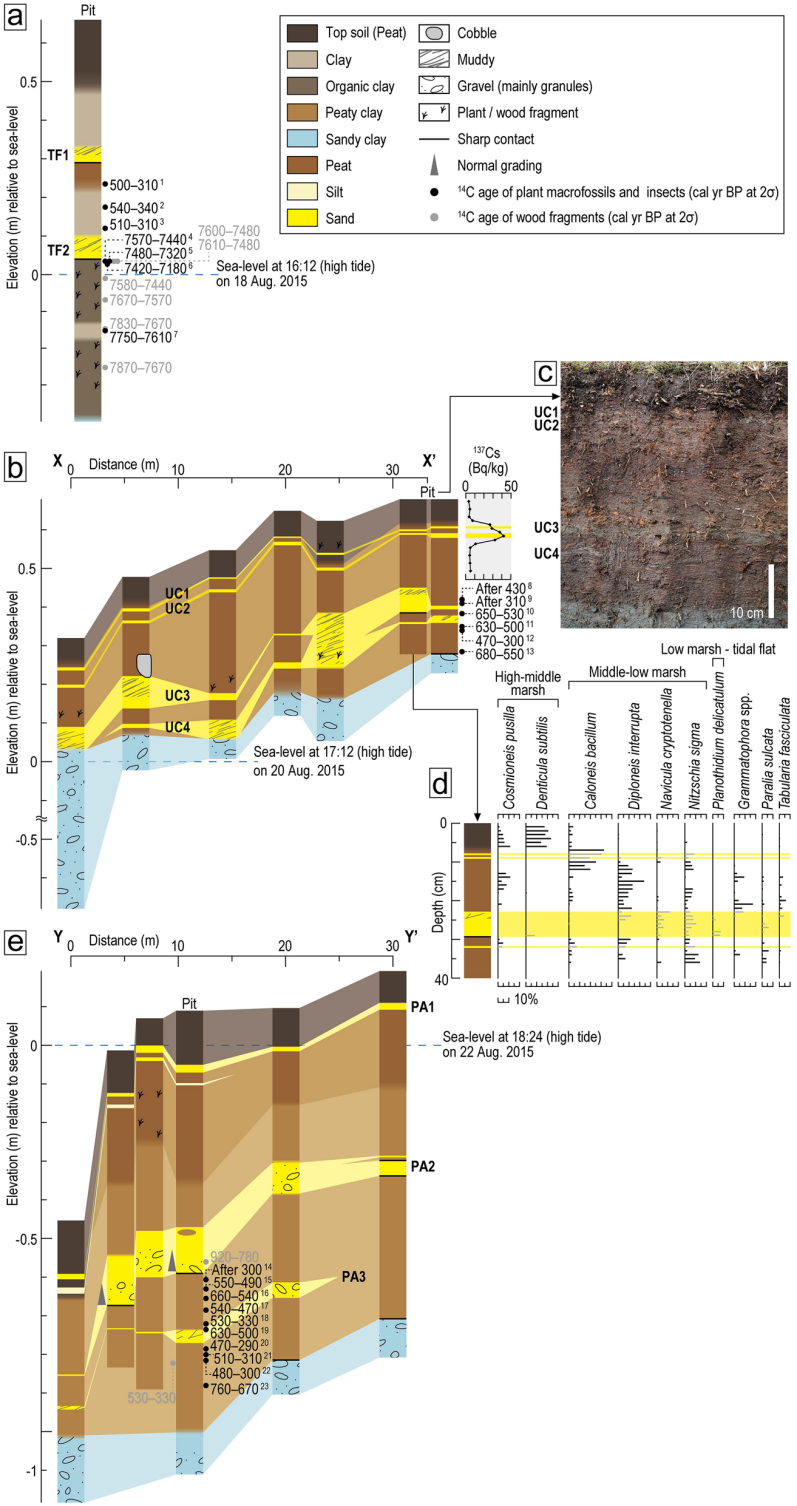
Stratigraphic evidence from study sites. (**a**) Tofino pit lithology (see Fig. 1b for location) and chronology. Details of radiocarbon ages are given in Supplementary Table S1. Numerical superscripts following radiocarbon ages correspond to the sample numbers in Fig. 3 and Table S1. (**b**) Stratigraphic cross-section along transect X-X’ in Ucluelet (see Fig. 1c for location) showing radiocarbon ages (Supplementary Table S1) and vertical distribution of ^137^Cs. Numerical superscripts following radiocarbon ages correspond to the sample numbers in Fig. 3 and Table S1. (**c**) Photograph of Ucluelet pit wall. (**d**) Relative abundance of selected brackish-marine diatom species from the Ucluelet pit. Gray bars indicate assemblages within sand sheets UC1–4 (full diatom assemblage in Supplementary Fig. S1). (**e**) Stratigraphic cross-section along transect Y-Y’ in Port Alberni (see Fig. 1d for location) showing radiocarbon ages (Supplementary Table S1). Numerical superscripts following radiocarbon ages correspond to the sample numbers in Fig. 3 and Table S1.

We excavated a pit (~ 1 m in depth) in a salt marsh along Browning Passage, ~ 7 km southwest of the community of Tofino (Fig. 1b). Here, the deposit consists of dark brown organic clay, sand (TF2); light olive-gray clay, peat, sand (TF1); light olive-gray clay; and a top soil (dark brown peat of the modern salt marsh) in ascending order (Figs. 2a and Supplementary Fig. S1a). The dark brown clay overlying bluish sandy clay at the base of the pit (~ 105 cm in depth) includes abundant plant and wood fragments. A ~ 5 cm-thick clay layer interbedded within the dark brown clay is less organic and shows light olive-gray color. The lower sand sheet, TF2, abruptly overlies the dark brown clay with an erosional basal contact. TF2 is composed of moderately-sorted fine gray sand that grades upwards to a muddy and patchy sediment. The thickness of TF2 varied from 2 to 6 cm on the pit wall. The upper contact boundary is relatively clear. TF2 is covered by a ~ 15 cm-thick light olive-gray clay with orange mottles that gradually changes upwards to peaty clay at a depth of around 44 cm below the surface. The upper sand sheet, TF1, is ~ 3 cm thick and abruptly overlies the peaty clay. TF1 has an erosional basal contact with the underlying peaty clay, and a relatively clear abrupt upper contact with the overlying light olive-gray clay including orange mottles. The light olive-gray clay gradually changes upwards into peat that typifies the modern salt marsh at a depth around 18 cm.

Loss on ignition (LOI), indicating organic content^37^, show relatively lower values in the sandy deposits and higher value in the organic units (Supplementary Fig. S1a). LOI values show a similar trend below and above each sand sheet, TF1 and TF2. LOI abruptly decreases from the dark brown clay to TF2 and gradually increases in the lower light olive-gray clay to the peaty clay succession. LOI shows an abrupt decrease again at the boundary between the peaty clay and overlying TF1 and then increases toward the ground surface.

**Figure 3:**
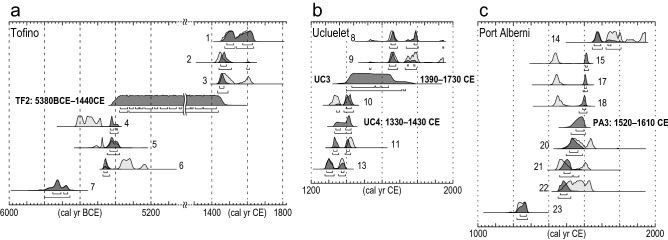
Estimates of the age of sand sheets TF2 (**a**), UC3 and UC4 (**b**), and PA3 (**c**). Ages of plant macrofossils and insects were used for age estimation. Constrained ages (2σ range) were calculated using OxCal 4.4^60,61^ with the IntCal20^62^. Age estimation followed the procedure of Lienkaemper and Bronk Ramsey (2009)^58^. Sample numbers correspond to the numerical superscripts shown in Fig. 2 and listed in Supplementary Table S1. Three anomalous ages, Nos. 12, 16, and 19, were excluded from the age estimation because they are younger or older than those obtained from horizons above and below them. The light and dark gray distributions indicate probability distribution functions (PDFs) of calibrated and modelled ages by OxCal, respectively. Horizontal bars below PDFs indicate 1σ and 2σ ranges of modelled ages.

The age of TF1 was estimated to be after 1450 CE based on older ages obtained from below TF1 (Fig. 2a, Supplementary Table S1). The age of TF2 was constrained by four older, and three younger radiocarbon ages (Figs. 2a, 3a and Supplementary Table S1). The constrained age of TF2 was 5380 BCE–1440 CE. Plant macrofossils obtained directly below TF2 provided ages older than 7000 cal yr BP, which indicates a hiatus between TF2 and the underlying organic clay. A thin white layer was identified ca 10 cm below TF2 on the peel (Fig. S1a). Based on ages below TF2, this layer may be the volcanic ash from Mt. Mazama that erupted ca 7600–7700 cal yr BP^38^.

Previous studies^13,32^ have reported similar successions of sand sheets and overlying mud to peat in salt marshes along Browning Passage. Based on sedimentological and foraminiferal analyses, each sedimentary succession has been attributed to coseismic subsidence, tsunami, and interseismic uplift associated with the 1700 CE Cascadia earthquake and an older event. Based on lithostratigraphy and chronology, the deposits observed in this study agree well with previous work^13,32^. Therefore, in this study, both TF1 and TF2 can be interpreted as tsunami deposits with the former being laid down by the 1700 CE Cascadia event.

#### Ucluelet

We collected four cores of 0.5–1 m length and excavated a ~ 0.5 m-deep pit along a ~ 30 m-transect in a salt marsh of Ucluelet Inlet (Fig. 1c). The deposit consists of sandy clay, peat, and a top soil (dark brown peat comprising the modern salt marsh) in ascending order (Fig. 2b,c). The bluish-gray sandy clay, consistent with the modern tidal flats, also includes some gravel (granules). It is more than 70 cm thick at the closest site to the tidal flat. The boundary with the overlying brown peat is relatively clear and sometimes sharp. The peat is 30–50 cm thick along the transect, thinning towards the tidal channel. The color of the peat, between about 13 to 21 cm deep, is darker with a high LOI indicating it is less muddy than the peats above and below (Supplementary Fig. S1b).

We found four continuous sand sheets within the peat along the transect (UC1, UC2, UC3, and UC4 in descending order). UC1 and UC2, UC3 and UC4 are interbedded in the uppermost and lower parts of the peat, respectively. UC3 and UC4 are composed of a very fine to fine gray sand, and are 0.5–8 cm thick. In some locations, only one sand sheet was evident in the lower part of peat. UC1 and UC2 are composed of a very fine gray sand, are less than 1 cm thick, and are within 13 cm of the ground surface. LOI values increase upward overall but show lower values near the sand sheets.

Fossil diatom assemblages in the peat and interbedded sand sheets record changes in the sedimentary environment over the past 700 years (Figs. 2d and Supplementary Fig. S3). Although brackish-marine species are dominant in all samples, their composition varies with depth. From 7 to 36 cm in depth, diatoms common in middle-low marshes such as *Diploneis interrupta* and *Nitzschia sigma*^39–41^ are predominant. Their proportion becomes higher above UC3, especially between 11 and 20 cm in depth. This section is also characterized by a dark brown peat and high LOI values (Figs. 2c and Supplementary S1b). In the top 6 cm, *Cosmioneis pusilla* and *Denticula subtilis*^39–41^ are abundant, representing the current high-middle marsh environment. Among the four sand sheets, only UC3 contained different species including *Navicula cryptotenella* and *Planothidium delicatulum* compared to those in the surrounding peat. *P. delicatulum* is generally common in low marshes and tidal flats^39–41^, and their presence here may indicate the seaward source of UC3.

Ages of UC1 and UC2 were inferred from ^137^Cs concentrations measured within the top 20 cm of the section (Fig. 2b). The ^137^Cs fallout peak indicating 1963 CE^42^ was found within UC2 at 9–10 cm in depth and therefore both UC1 and UC2 were deposited around 1963 CE. It is difficult to distinguish each age precisely because both UC1 and UC2 are dispersed over a few centimeters of the pit wall. UC3 and UC4 ages were constrained by five radiocarbon ages obtained from below UC4, between UC3 and UC4, and above UC3 (Figs. 2b, 3b and Supplementary Table S1). The constrained ages for UC3 and UC4 are 1390–1730 CE and 1330–1430 CE, respectively.

Previous studies^13,32^ have found three sand sheets within the mud and peat from salt marshes along Ucluelet Inlet. Both of the lower two of these sand sheets were overlain by a sharp contact with mud to peat sequences. This sedimentary succession has been correlated with that in Tofino, and therefore attributed to tsunami and seismic deformation^13,32^. Stratigraphic correlation between the present study and previous ones is complicated because not only are there differences in the number and stratigraphic position of the sand sheets, but also no dating was carried out in the earlier work. Based on ^137^Cs dating, UC1 and/or UC2 were most likely deposited by the 1964 Alaska tsunami which also affected Tofino^13^ and Port Alberni^36^. The age of UC3 was estimated to be 1390–1730 CE, its broad range was increased by the plateau on the radiocarbon calibration curve. UC3 appears most likely to have been deposited by the 1700 CE Cascadia tsunami since it matches well with geological evidence reported across much of the southwest coast of Vancouver Island^27,29,31,35^. Based upon its age, sedimentological characteristics, and fossil diatom assemblage, we are unable to be definitive about the deposition process for UC4. However, the geomorphological setting of the study site eliminates a possible flood origin. Therefore, UC4 originated from either a tsunami or storm surge. Although the landward extent of event deposits might be one criterion to distinguish between tsunami and storm deposits, it does not apply to this site.

#### Port Alberni

We collected five cores of 0.7–1 m length and excavated a ~ 1.2 m-deep pit along a ~ 30 m-transect in a salt marsh near the Somass River, at the head of Alberni Inlet (Figs. 1d,e, and Supplementary Fig. S1c). Here, the deposit consists of sandy clay, peaty clay, peat, and a dark brown peat top soil (representing the modern salt marsh) in ascending order (Fig. 2e). The bluish-gray sandy clay, which continues to modern tidal flat sediments, includes medium sand to granules with some pebbles. Its upper boundary with the overlying peaty clay is gradual on the pit wall, but sharp at inland core locations. The gray-brown peaty clay becomes less muddy up-section, and gradually changes to a brown peat between 30 and 50 cm below the ground surface. The peaty clay to peat succession, including the top soil, is 40–100 cm thick along the transect, becoming thinner toward the seaward edge of the salt marsh.

Three continuous sand sheets were recognized within this succession along the transect (PA1, PA2, and PA3 in descending order). PA3 is composed of a very fine to fine sand, and is indistinct in the three most seaward locations but ~ 4 cm thick in the pit. It is absent at the most inland location. PA2 is composed mainly of medium sand. It varies between 1 and 13 cm thick. On the pit wall, PA2 has an erosional basal contact with the underlying peaty clay. It contains granules in the lowest few centimeters, and fines upwards. It also contains mud clasts in the upper part of the unit. PA1 was found as a thin discontinuous layer at the boundary between the peat and top soil. It is composed of fine to medium olive-gray sand, and is less than 2 cm thick. A faint silt layer of less than 1 cm thick was observed directly below PA1 at the four most seaward locations. LOI show relatively lower values in PA1 and PA2 than in the peaty clay and peat (Supplementary Fig. S1c). LOI values continue to increase gradually between PA1 and PA2, PA2 and PA3.

The age of PA2 was estimated to be after 1650 CE, based on older radiocarbon ages obtained from below PA2 (Fig. 2e, Supplementary Table S1). The age of PA3 was constrained by four older and four younger radiocarbon ages (Figs. 2e, 3c and Supplementary Table S1) to between 1520 and 1610 CE.

Three ubiquitous sand sheets have been recognized within mud and peat sequences beneath salt marshes near the mouth of Somass River in previous studies^13,32^. Based on ^137^Cs analysis, eyewitness account, and its distribution area, the uppermost sheet was inferred to have been deposited by the 1964 Alaska tsunami. The middle sand sheet has been correlated to the 1700 CE Cascadia tsunami^13,32^. Based on their ages and stratigraphic positions, PA1 and PA2 have correlated with these upper and middle sand sheets, respectively. There is not a specific counterpart for layer PA3 since it appears to have been deposited > 300 years later (and at a stratigraphically shallower position) than the lowest sand sheet in previous studies. It is therefore difficult to determine the origin of PA3 among extreme waves including tsunamis, storm surges, and floods, based upon its age, sedimentological characteristics, and geomorphological setting.

### Inter-regional correlation with paleoseismic evidence along the Cascadia Subduction Zone

We examined sand sheets interbedded within peat and mud deposited under the low-energy coastal environment of southwestern Vancouver Island. Sedimentological and chronological analyses, and the comparison with previous studies^13,32^, allow the following interpretation about the origins of event deposits: (1) UC1 and/or UC2, and PA1 were most likely deposited by the 1964 Alaska tsunami. (2) TF1, UC3, and PA2 by the 1700 CE Cascadia tsunami. Although the age of UC3 (1390–1730 CE) has a wide range, it seems likely that it too was laid down by the 1700 CE event.

The new chronology established for this paper provides better constraints for the ages of extreme events that occurred prior to the 1700 CE Cascadia earthquake. Though we cannot rule out storms and floods from the origins of UC4 and PA3, we focus on the possibility of their tsunami origin in this section. Correlative beds of UC4 were not found in Port Alberni, similarly, those of PA3 were not in Ucluelet. These facts do not necessarily eliminate their tsunami origin. For example, if a tsunami had affected both sites, the tsunami behavior is likely to have varied due to different geomorphological settings between Ucluelet and Port Alberni. A tsunami wave with enough energy to emplace a deposit may have reached only one of the two sites. Moreover, even if tsunami deposits had been laid down at both sites, it is entirely plausible that these may have been disturbed or eroded by post-depositional processes. The age of TF2 (5380 BCE–1440 CE) partly overlaps with that of UC4 (Fig. 3a,b). However, we can not determine whether UC4 and TF2 were deposited by the same event because the limiting maximum age of TF2 is not well constrained.

**Figure 4:**
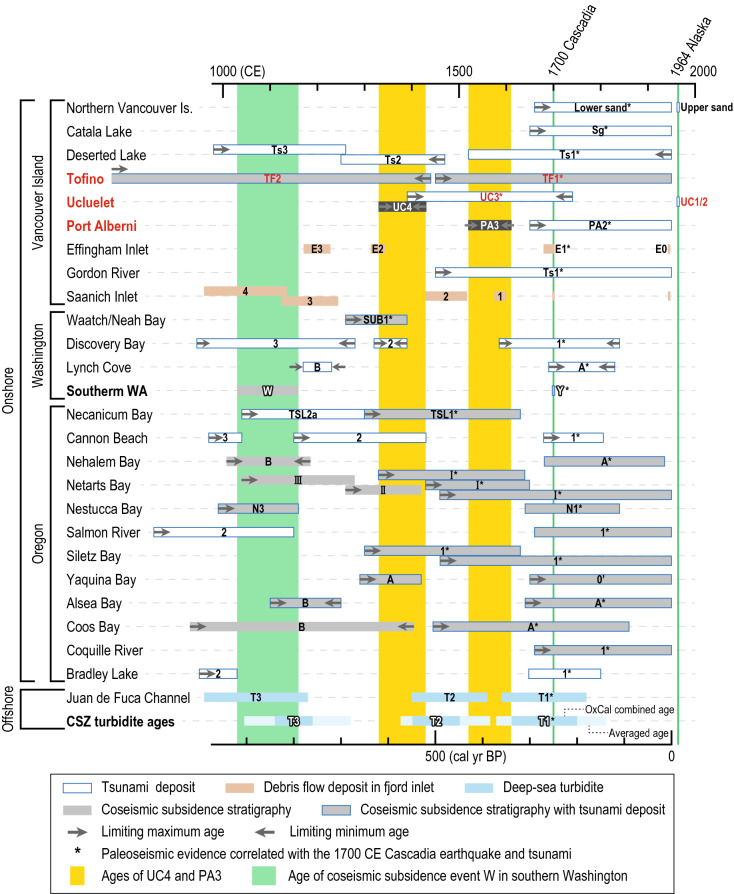
Comparison of age estimates for paleoseismic evidence along the Cascadia subduction zone over the past 1000 years: Locations listed from north to south—Northern Vancouver Island^27,33^ (Koprino Habour, Neroutsos Inlet, and Fair Harbour), Catala Lake^34^, Deserted Lake^27,35^, Gordon River^27^, Effingham Inlet^30,45^, Saanich Inlet^74,75^, Waatch/Neah Bay^46^, Discovery Bay and Lynch Cove^43,44^, Southern Washington^9,28^ (Copalis River, Grays Harbor, Willapa Bay, and Columbia River), Necanicum River^48,51^, Cannon Beach^47^, Nehalem Bay^48,51,76^, Netarts Bay^48,77^, Nestucca Bay^78^, Salomon River^15^, Siletz Bay^48,52^, Yaquina Bay^49,79,80^, Alsea Bay^10^, Coos Bay^50^, Coquille River^20^, Bradley Lake^81^, Juan de Fuca channel, and CSZ averaged turbidite ages^18^ (locations shown in Fig. 1a) Referenced data are compiled in Table S2. Data from California are not included because there is no paleoseismic evidence correlated with UC4 and PA3.

We discuss the correlation with paleoseismic evidence from other regions along the CSZ based on the timing of UC4 (1330–1430 CE) and PA3 (1520–1610 CE) (Figs. 1a and 4, Supplementary Table S2). There is correlative tsunami deposit dating evidence for UC4 from Vancouver Island and northern Washington at Deserted Lake^35^ and Discovery Bay^43,44^, and a debris flow from Effingham Inlet^30,45^ (Fig. 4). The event age for Discovery Bay^43,44^ was constrained by limiting maximum and minimum ages of growth-position *Triglochin maritima* leaf-bases. Although the evidence from Waatch/Neah Bay^46^ has been correlated with the 1700 CE tsunami, its age overlaps with that of UC4. We found no synchronous evidence in the well-documented coastal wetland sites of southern Washington that provide a rigorous paleoseismic history mainly based on tree ring dating^9,21,22^. Here, only two coseismic subsidence events, Y and W, regarded as the 1700 CE and penultimate Cascadia earthquakes, respectively, are reported for the past millennium^28^. However, the ages of UC4 and PA3 lie between these two most recent events in southern Washington. In Oregon, the age of a tsunami deposit from Cannon Beach^47^ partly overlaps with that of UC4, with correlative evidence of coseismic subsidence also reported from Netarts Bay^48^, Yaquina Bay^49^, and Coos Bay^50^. Evidence from Necanicum River^48,51^ and Siletz Bay^48,52^, which has been correlated with the 1700 CE earthquake in previous studies, could also be correlated with UC4. Overall, the geographical distribution of synchronous onshore evidence with UC4 includes Vancouver Island, northern Washington, and northern Oregon. Albeit some potential coeval evidence with PA3 was also found in the same area such as Deserted Lake^35^, Gordon River^27^, Necanicum River^48,51^, Netarts Bay^48^, Siletz Bay^48,52^, and Coos Bay^50^, all have previously been attributed to the 1700 CE earthquake. A comparison with offshore turbidites^18^ indicates that UC4 could be correlated with T2, which has been proposed as a full-margin rupture event prior to the 1700 CE earthquake (turbidite T1) (Fig. 4). T2 has been documented along much of the CSZ except for its northern end. A recent review^31^ suggested that some correlative onshore evidence may exist in northern Washington and Vancouver Island. Most of the few candidate onshore deposits are ~ 100 years older than T2^18,27,53^, although they might be potential correlatives, considering that turbidite ages are affected by temporal and spatial variability of reservoir ages^18^. Unlike most onshore paleoseismic evidence though, UC4 does partly overlap with that of T2.

The geographical distribution of paleoseismic activity about 600–700 years ago including turbidite T2 supports their tsunami origin suggested by the recent review^31^. Assuming that UC4 was left by a tsunami, there could be three candidate source earthquakes based on the possible inter-regional correlations described above (Fig. 4). First, an earthquake that ruptured the northern CSZ may have generated a tsunami affecting only Vancouver Island and northern Washington^31^. This is supported by existing research that has found no evidence for coseismic subsidence and tsunamis in southern Washington over the age range for UC4. This suggests that UC4 was deposited by a relatively small earthquake that only ruptured the northern segment of the CSZ off Vancouver Island. We note that no robust synchronous turbidite has been found in the last 1500 years at Barkley Canyon facing the southwest coast of Vancouver Island^18^ (Fig. 1a). A decrease in sediment supply in the late Holocene was documented at Slipstream Slump, 30 km northwest of Barkley Canyon^54^. Second, UC4 may have been the result of a small magnitude penultimate full-margin earthquake, synchronous with turbidite T2. Correlative paleoseismic evidence from northern Oregon supports this hypothesis. Whereas the timings of all seven coseismic subsidence events in southern Washington^28^ are consistent with those of turbidites T1, T3–T8 over the last 4000 years, there is no onshore counterpart for T2^18^ (Fig. 4). Indeed, some of the trees in southern Washington which died as a result of subsidence associated with the 1700 CE earthquake, were not affected by any event occurring during the age range of UC4^22,43,53^. Therefore, it is possible that the penultimate Cascadia earthquake may have been smaller in magnitude, and did not result in any sizeable coseismic subsidence or produce a tsunami large enough to be preserved in many coastal regions along the CSZ. Third, a remote tsunami might be a candidate source. Two coeval tsunami deposits have been documented in the Aleutian Islands (Sedanka: S3^55^, Umnak: C2^56^), and as reported for the 1964 Alaska earthquake^36^, Aleutian megathrust events could generate tsunamis affecting the west coast of Vancouver Island^31,57^.

A new chronology for event deposits prior to the 1700 CE Cascadia tsunami is proposed based upon evidence reported from the southwest coast of Vancouver Island. The proposed ages are not consistent with the age of the penultimate great Cascadia earthquake inferred from onshore paleoseismic evidence. On the other hand, the age of the penultimate Cascadia earthquake inferred from offshore turbidites partly overlaps with the new ages. The newly constrained ages from Vancouver Island are an important addition to our understanding of the timing of the predecessor of the 1700 CE Cascadia earthquake. Moreover, our study highlights the importance of reassessing the chronology of paleoseismic evidence along the west coast of Vancouver Island. Further studies are needed to help resolve the discrepancy in the timing of the penultimate full-margin Cascadia earthquake between onshore and offshore paleoseismic evidence.

## Methods

### Sampling and sedimentological analysis

We examined the stratigraphy beneath salt marshes by using a gouge core (2.5-cm in diameter, up to 1 m in depth) and excavating pits. Block samples for analyses were collected from pit walls. We made sediment peels to observe sedimentary structures in detail. Peels were collected from pit walls and block samples using a hydrophilic grout^58^. Peels highlight the differences between sand and mud/peat. Because the grout penetrates coarse sediment deeper than fine sediment, which results in a change in the thickness of peels (Photographs in Supplementary Fig. S1). Samples were also scanned using computed tomography (CT) (Supria Grande, Hitachi) at the Geological Survey of Japan (Fig. S1).

### Dating and age estimation of sand sheets

We used a combination of ^14^C and ^137^Cs dating to constrain the ages of sand sheets. For radiocarbon dateable material, using a binocular microscope, we sampled insects and plant macrofossils, including fruits, cones, leaves, needles, and moss from sediment samples. In addition, we also used wood fragments, including twigs, fir and bark picked out from pit walls, as dating material. Samples were dated by accelerator mass spectrometry at Beta Analytic, Florida, USA and DirectAMS, Washington, USA. The ages for sand sheets, TF2, UC3, UC4, and PA3, were constrained using dates from insects and plant macrofossils (No. 1–11, 13–15, 17, 18, 20–23 in Supplementary Table S1). Dating samples were obtained from below and above the sand sheets, indicating maximum and minimum ages, respectively. According to the age estimation procedure of Lienkaemper and Bronk Ramsey (2009)^59^, we used Bayesian statistics in the radiocarbon calibration program OxCal 4.4^60,61^ with the IntCal20 radiocarbon calibration dataset^62^ (OxCal code in Supplementary Fig. S4). Three anomalous ages, Nos. 12, 16, and 19, were excluded from the age estimation of UC3–4 and PA3 because they were either younger or older than ages obtained from horizons above and below them. Although the reason for the reversals of radiocarbon ages is uncertain, it might be attributed in part to bioturbation.

^210^Pb and ^137^Cs were analyzed in 14 samples from Ucluelet. Samples were collected at intervals of 1–2 cm from 0 to 19 cm in depth. In each case, the homogenized sample of 3–5 g was placed into a centrifuge tube. After a month, the tube was positioned on a well-type germanium semiconductor detector (GCW2022, Canberra Industries Inc., Connecticut, USA) to determine the count rate of gamma ray emitted from ^137^Cs, ^210^Pb, and ^214^Pb. The count rate was converted to an activity by using a diluted New Brunswick Laboratory (NBL) counter calibration sample and The Japan Society for Analytical Chemistry (JSAC) certified reference material, considering the detection efficiency on the height of the sample. Before the measurement of the unknown sample, we measured Geological Survey of Japan (GSJ) geochemical reference sample to examine our calibration for a part of Pb activity.

### Fossil diatom analysis

Fossil diatoms were analyzed in 36 samples from the pit at Ucluelet. Samples were obtained at intervals of 1 cm, between 1 and 36 cm in depth. The collected samples were prepared following Sawai et al. (2016)^41^ for identifying and counting diatoms. At least 250 diatom valves were counted under a light microscope for each prepared slide. Diatom identification and ecological interpretations followed standard references^39–41,63–72^.

### Loss on ignition

Organic content was measured by loss on ignition. Samples were collected at 1 cm intervals from 0 to 103 cm in depth at Tofino, from 5 to 45 cm in depth at Ucluelet, and from 0 to 98 cm in depth at Port Alberni. The dried sample was ignited at 550 °C for 4 h. Organic content was calculated as a percentage of weight loss of the dried sample after heating at 550 °C.

## Supplementary Information

Supplementary Information.

## Data Availability

The datasets used and/or analyzed during the current study available from the corresponding author on reasonable request.
