# Supplementary material for: A new chronology for tsunami deposits prior to the 1700 CE Cascadia earthquake from Vancouver Island, Canada
Source: Sci Rep. 2022 Jul 22;12:12527. doi: 10.1038/s41598-022-16842-8 (PMC9307600; doi:10.1038/s41598-022-16842-8)
Supplement: Supplementary file 1 — Supplementary Information. [file 41598_2022_16842_MOESM1_ESM.pdf]

Supplementary Information

A new chronology for tsunami deposits prior to the 1700 CE Cascadia earthquake from Vancouver Island, Canada

Koichiro Tanigawa[1]*, Yuki Sawai[1], Peter Bobrowsky[2], David Huntley[3], James Goff[4, 5], Tetsuya Shinozaki[1], Kazumi Ito[1]

[1]*Geological Survey of Japan, National Institute of Advanced Industrial Science and Technology (AIST), Tsukuba, Ibaraki 305-8567, Japan*

[2]*Geological Survey of Canada, Natural Resources Canada, Sidney, British Columbia V8L 4B2, Canada*

[3]*Geological Survey of Canada, Natural Resources Canada, Vancouver, British Columbia V6B 5J3, Canada*

[4]*School Biological, Earth and Environmental Sciences, University of New South Wales, Sydney, New South Wales 2052, Australia*

[5]*School of Ocean and Earth Science, University of Southampton, UK*

*Corresponding author

Koichiro Tanigawa

Geological Survey of Japan, National Institute of Advanced Industrial Science and Technology (AIST), Site C7, 1-1-1 Higashi, Tsukuba, Ibaraki 305-8567, Japan

Email: k-tanigawa@aist.go.jp

Caption for supplementary figures and tables

Supplementary Figure S1

Photographs of pit walls and peels, CT images, and loss on ignition (LOI) for deposits obtained from the pits: (a) Tofino, (b) Ucluelet and (c) Port Alberni. The peels and CT images of each site were taken on different sections of samples collected from the same pits.

Supplementary Figure S2

Lithology and ^{14}C ages from the pits in (a) Tofino and (b) Ucluelet in the 2016 field survey). Locations of the pits are almost the same as those in the 2015 field survey (Figs. 1b and 1c).

Supplementary Figure S3

Diatom assemblages from the pit in Ucluelet. Diatom species within sand sheets UC1–4 are indicated by gray bars.

Supplementary Figure S4

OxCal code for the age estimations of TF2, UC3, UC4, and PA3.

Supplementary Table S1

Radiocarbon ages from Tofino, Ucluelet, and Port Alberni.

Calibrated ages were calculated using the radiocarbon calibration program OxCal 4.4[1,2] with the IntCal20 radiocarbon calibration data set[3].

Supplementary Table S2

Age estimates for paleoseismic evidence cited in this paper (Fig. 4).

Fig. S1

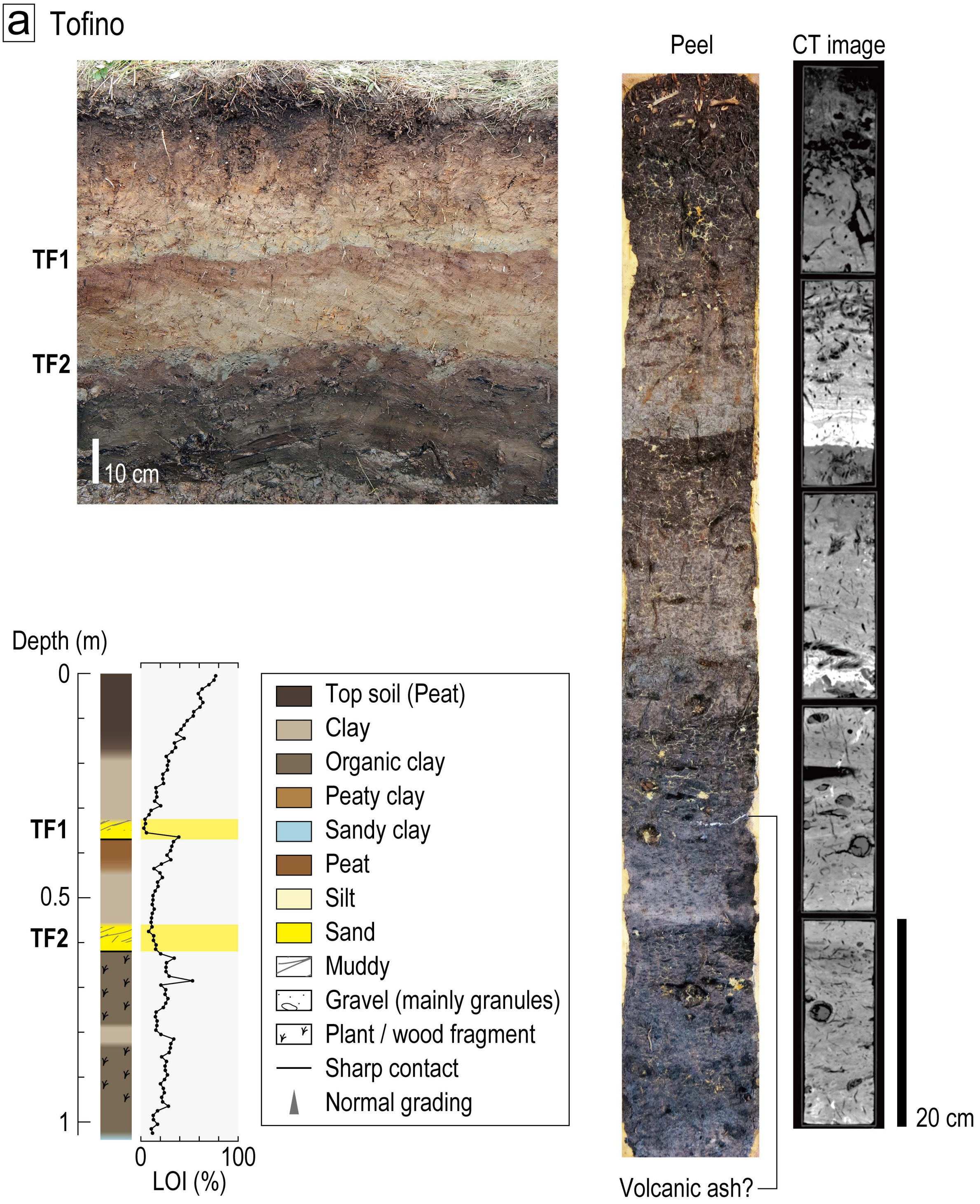
a
Tofino
TF1
TF2
10 cm
Depth (m)
0
0.5
1
TF1
TF2
0
100
LOI (%)
Top soil (Peat)
Clay
Organic clay
Peaty clay
Sandy clay
Peat
Silt
Sand
Muddy
Gravel (mainly granules)
Plant / wood fragment
Sharp contact
Normal grading
Peel
CT image
20 cm
Volcanic ash?

Fig. S1 *(continued)*

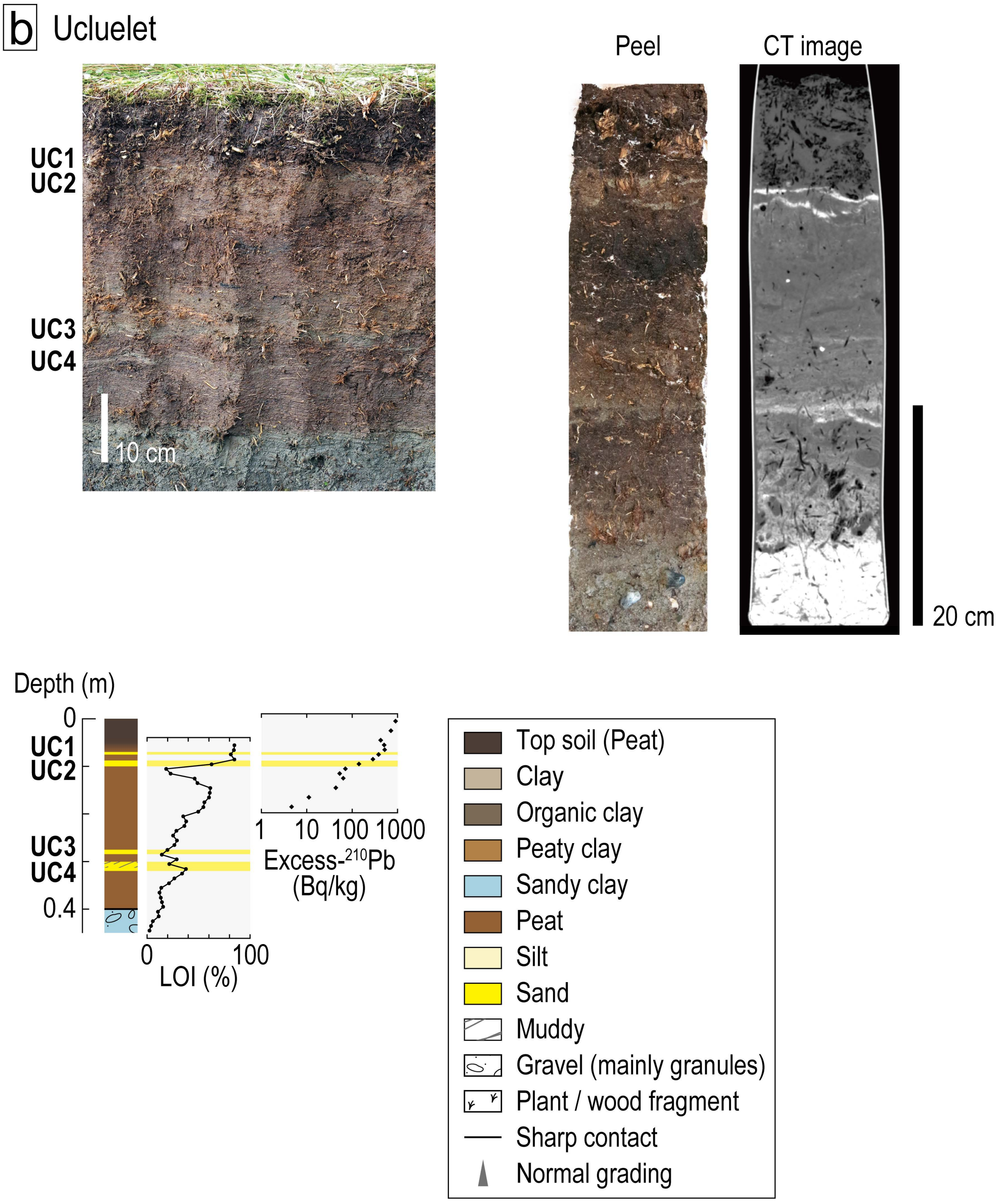

Fig. S1 *(continued)*

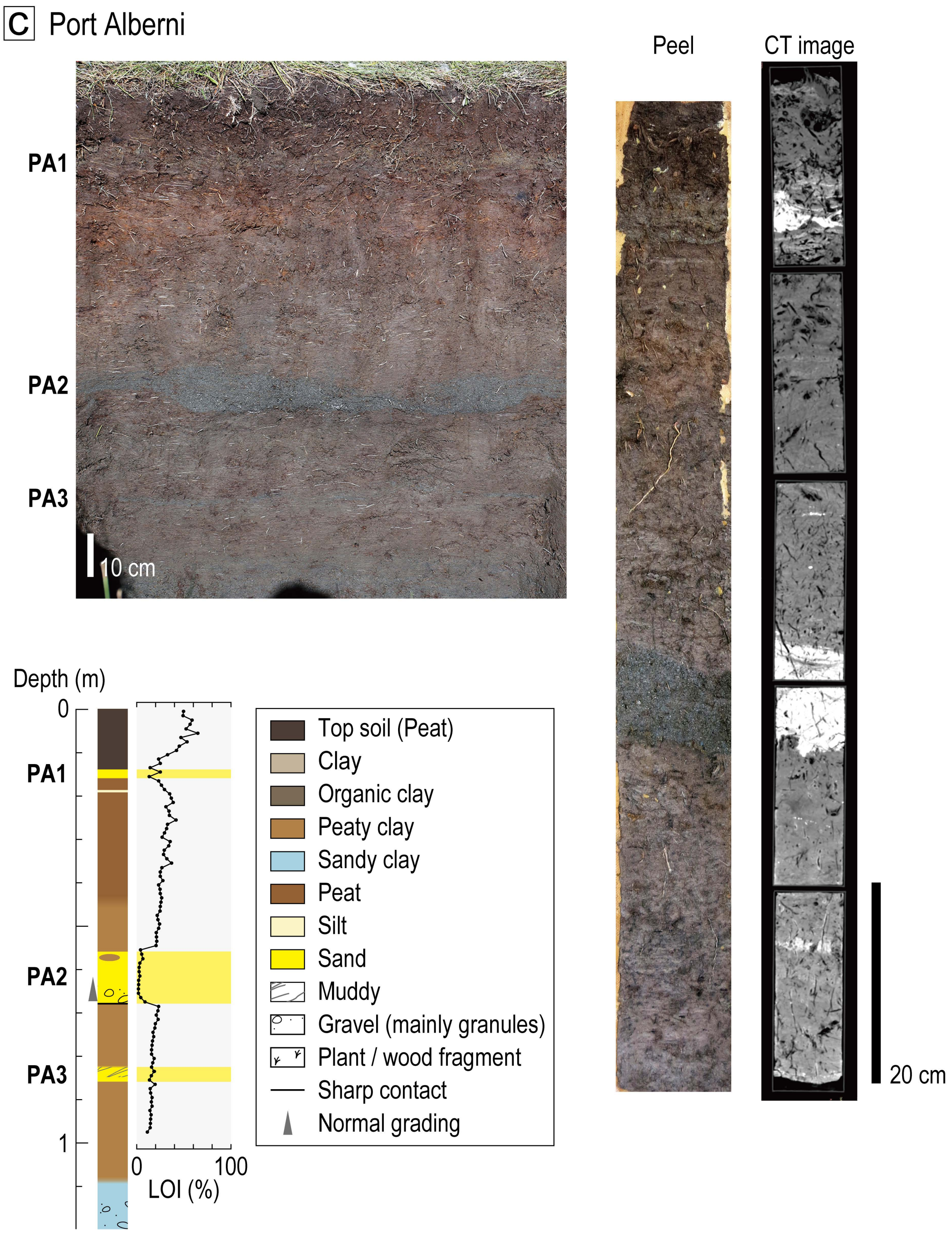

Fig. S2

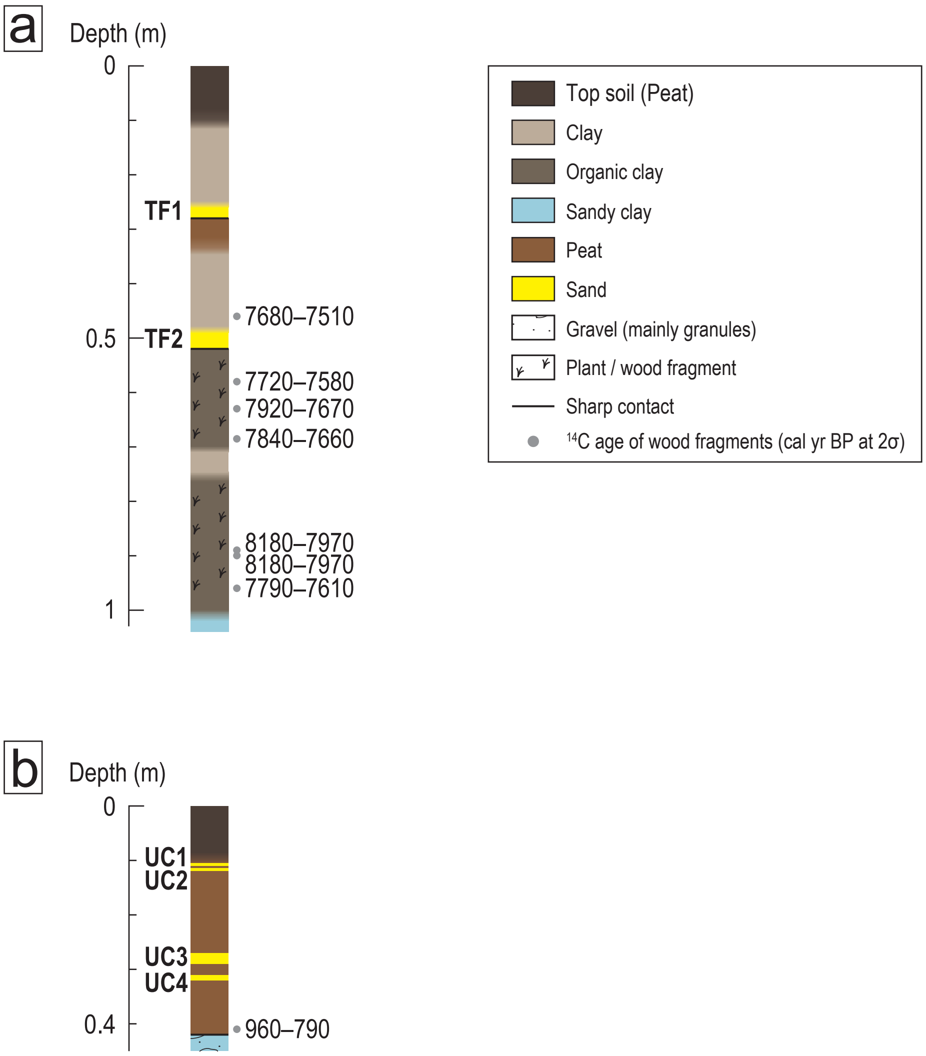
a
Depth (m)
0
0.5
1
TF1
TF2
7680–7510
7720–7580
7920–7670
7840–7660
8180–7970
8180–7970
7790–7610
Top soil (Peat)
Clay
Organic clay
Sandy clay
Peat
Sand
Gravel (mainly granules)
Plant / wood fragment
Sharp contact
14C age of wood fragments (cal yr BP at 2σ)
b
Depth (m)
0
0.4
UC1
UC2
UC3
UC4
960–790

Fig. S3

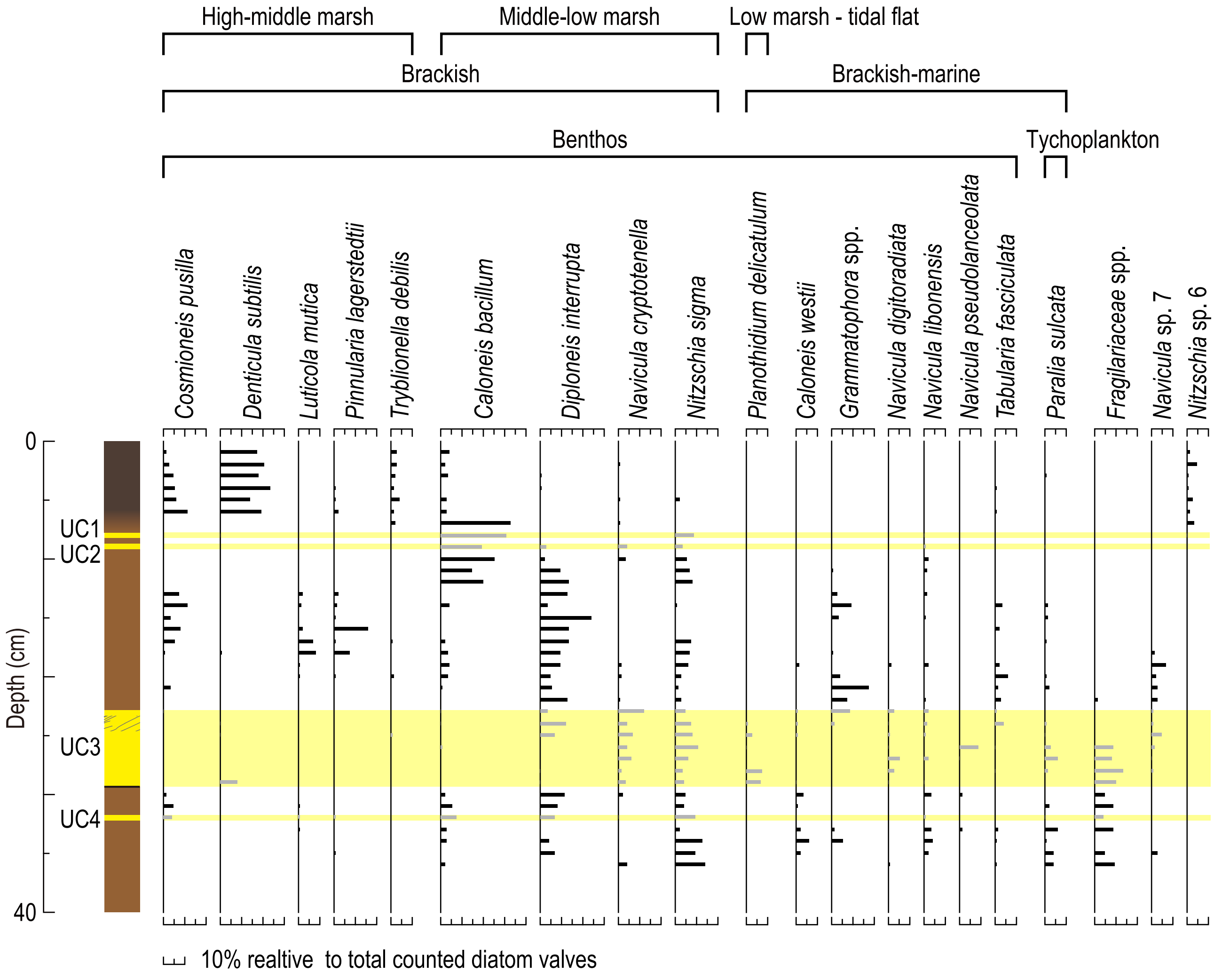

High-middle marsh
Middle-low marsh
Low marsh - tidal flat
Brackish
Brackish-marine
Benthos
Tychoplankton
Cosmioneis pusilla
Denticula subtilis
Luticola mutica
Pinnularia lagerstedtii
Tryblionella debilis
Caloneis bacillum
Diploneis interrupta
Navicula cryptotenella
Nitzschia sigma
Planothidium delicatulum
Caloneis westii
Grammatophora spp.
Navicula digitoradiata
Navicula libonensis
Navicula pseudolanceolata
Tabularia fasciculata
Paralia sulcata
Fragilariaceae spp.
Navicula sp. 7
Nitzschia sp. 6
Depth (cm)
0
40
UC1
UC2
UC3
UC4
10% realtive to total counted diatom valves

Fig. S4

OxCal code

TF2

```
Plot()
 {
  Sequence("Tofino")
  {
   Boundary("base");
   R_Date("CM1_80-81", 6850, 30);
   R_Date("CM3_61cone", 6360, 30);
   Phase("CM3_60-61")
   {
    R_Date("CM3_60-61moss", 6500, 30);
    R_Date("CM3_60-61leaves", 6620, 30);
   };
   Date("TF2");
   R_Date("CM3_53-55", 390, 30);
   R_Date("CM1_48-49", 440, 30);
   R_Date("CM1_42-43", 350, 30);
   Boundary("top");
  };
 };
```

Fig. S4 *(continued)*

UC3 and UC4

```
Plot()
 {
  Sequence("Ucluelet")
  {
   Boundary("base");
   R_Date("38-40", 660, 30);
   R_Date("32-33", 510, 30);
   Date("UC4");
   R_Date("UC-2 23-24", 570, 30);
   Date("UC3");
   R_Date("26-27", 210, 30);
   R_Date("25-26", 240, 30);
   Boundary("top");
  };
 };
```

Fig. S4 *(continued)*

PA3

```
Plot()
 {
   Sequence("Port Alberni")
   {
     Boundary("base");
     R_Date("96-98", 790, 30);
     R_Date("90-91", 330, 30);
     R_Date("88-90", 390, 30);
     R_Date("87-88", 300, 30);
     Date("PA3");
     R_Date("80-82", 430, 30);
     R_Date("76-79", 450, 30);
     R_Date("71-73", 470, 30);
     R_Date("69-70", 180, 30);
     Boundary("top");
   };
 };
```

Table S1

Table S1. *Radiocarbon ages from Tofino, Ucluelet, and Port Alberni.*

Calibrated ages were calculated using the radiocarbon calibration program OxCal 4.4[1, 2] with the IntCal20 radiocarbon calibration data set[3].

Site No.*	Position	Material	Conventional age (yr BP)	Calibrated age (cal yr BP, rounded to nearest 10 yrs) 1σ range	2σ range	Lab no.
**Tofino**						
1		Fruits	350 ± 30	480–420, 380–320	490–310	Beta-482392
2	Above TF2	Fruits	440 ± 30	520–480	540–450, 350-340	Beta-482393
3		Fruits	390 ± 30	500–440, 360–330	510–420, 380–310	Beta-428330
		Thuja plicata[†]	6748 ± 45	7660–7570	7680–7560, 7540–7510	D-AMS 018070
4		Leaves	6620 ± 30	7570–7530, 7520–7470, 7440–7430	7570–7430	Beta-448113
5		Moss	6500 ± 30	7430–7410, 7390–7330	7480–7320	Beta-448112
		Tsuga heterophylla	6674 ± 35	7580–7500	7610–7470, 7440–7430	D-AMS 105475
		Thuja plicata	6670 ± 33	7580–7500	7610–7470	D-AMS 105476
6		Cone (single grain)	6360 ± 30	7320–7250	7420–7390, 7340–7240, 7210–7160	Beta-423993
		Tsuga heterophylla	6630 ± 33	7570–7530, 7520–7470	7580–7430	D-AMS 105476
		Thuja plicata	6752 ± 35	7660–7630, 7620–7570	7680–7570, 7530–7520	D-AMS 105478
	Below TF2	*Thuja plicata*	6906 ± 36	7780–7680	7840–7660	D-AMS 105479
7		Fruits	6850 ± 30	7710–7650, 7640–7610	7750–7600	Beta-428331
		Thuja plicata	6919 ± 38	7790–7690	7840–7670	D-AMS 105480
		Thuja plicata[†]	6825 ± 34	7690–7610	7720–7580	D-AMS 018071
		Thuja plicata[†]	6936 ± 46	7830–7810, 7800–7690	7920–7900, 7860–7670	D-AMS 018072
		Thuja plicata[†]	6896 ± 34	7760–7670	7840–7810, 7800–7660	D-AMS 018073
		Root (*Alnus sp.*)[†]	7259 ± 52	8170–8080, 8050–8010	8180–7970	D-AMS 018074
		Root (*Alnus sp.*)[†]	7249 ± 38	8170–8080, 8050–8010	8180–7970	D-AMS 018075
		Root (*Alnus sp.*)[†]	6858 ± 38	7740–7660, 7640–7620	7790–7610	D-AMS 018076
**Ucluelet**						
8	Above UC3	Needles	240 ± 30	320–280, 170–150	430–390, 320–260, 220–140, 30–0	Beta-480737
9		Needles	210 ± 30	300–270, 210–150	310–260, 230–140, 30–0	Beta-465206
10	Between UC3 and UC4	Fruits	570 ± 30	630–590, 560–540	650–580, 570–520	Beta-480738
11		Fruits, Needles	510 ± 30	550–510	630–610, 560–500	Beta-448036
12	Below UC4	Needles	320 ± 30	440–360, 330–310	470–300	Beta-476061
13		Fruits	660 ± 30	670–630, 590–560	680–620, 600–550	Beta-478272
		Twig (Picea sp.)[†]	991 ± 32	960–900, 870–820, 810–800	960–890, 880–790	D-AMS 018069
**Port Alberni**						
	Within PA2	Fir (*Picea* sp.)	923 ± 25	910–840, 830–780	920–770, 760–740	D-AMS 105482
14		Fruits, Leaves	180 ± 30	290–260, 220–140, 30–0	300–250, 230–130, 120–60, 40–0	Beta-439937
15		Insects	470 ± 30	530–500	550–490	Beta-490588
16	Above PA3	Moss	610 ± 30	650–580, 570–550	660–540	Beta-482394
17		Fruits	450 ± 30	520–490	540–470	Beta-491785
18		Fruits	430 ± 30	520–470	530–450, 360–330	Beta-468405
19		Insects, Fruits	510 ± 30	550–510	630–610, 560–500	Beta-440362
20		Fruits	300 ± 30	430–370, 330–300	460–290	Beta-448035
21		Fruits	390 ± 30	500–440, 360–330	510–420, 380–310	Beta-465205
22	Below PA3	Fruits (*Carex* spp.)	330 ± 30	450–350, 340–310	480–310	Beta-476060
		Bark (*Abies* sp.)	426 ± 26	510–470	530–450, 350–330	D-AMS 105483
23		Fruits	790 ± 30	730–680	740–670	Beta-482395

*Sample numbers 1–23 correspond to numbers in Figs 2 and 3.

[†]Samples collected in the 2016 field survey. Sampled positions are presented in Fig. S2. Unlabeled samples were collected in the 2015 field survey.

Table S2

Table S2 *Age estimates for paleoseismic evidence cited in this paper (Fig. 4).*

Site	Name	Radiocarbon age (yr BP)	Calender age (cal yr CE)	Details of dating (materials for radiocarbon dating, methods for age estimation)	Correlation in previous studies	Reference
Northern Vancouver Is.						
Koprino Harbour	lower sand	161±16	After 1660*	Leaf bases of *Triglochin maritimum* within the lower sand.<br>*T. maritimum* was living on the marsh and most closely date the time of sand deposition.	1700 CE Cascadia earthquake	Benson et al. (1997)[4]<br>Hutchinson and Clague (2017)[5]
Fair Habour	upper sand	N/A	N/A	Peat just below the upper sand indicated high concentrations of ^{137}Cs.	1964 CE Alaska earthquake	
Catala Lake	Sg<br>(sand-gravel sheet)	170±40	After 1650*	Maximum age†: Plant detritus capping Sg<br>170±40 yr BP is the youngest one among ^{14}C ages from plant detritus. The age was regarded as the maximum age for the time of deposition of Sg, based on the interpretation detritus layer and the subjacent Sg were deposited during the same event.	1700 CE Cascadia earthquake	Clague et al. (1999)[6]
Dserted Lake	Ts1 (core 97-109)	190±60	After 1520*	Minimum age†: Twig with needles of spruce above Ts1<br>The stratigraphic position is based on Fig. 10A in Hutchinson and Clague (2017)[5]	1700 CE Cascadia earthquake	Hutchinson et al. (2000)[7]<br>Hutchinson and Clague (2017)[5]
	Ts2 (core 97-109)	590±90	1230–1480*	Minimum age†: Hemlock needles from about 0.3 m above Ts2	Turbidite T2	Samples were collected by Hutchinson et al. (2000)[7],<br>but data for core 97-109 were unpublished.
	Ts3 (core 97-109)	930±80	980–1270*	Maximum age†: Hemlock needles from a coarse plant layer immediately beneath Ts3	Buried soil W	
Gordon River	Ts1	No data	1448–1949	Maximum age†: A wood fragment from immediately below Ts1	1700 CE Cascadia earthquake (likely)	Hutchinson and Clague (2017)[5]
Effingham Inlet	Seismite E0	—	1946	Event age was estimated by varve counting and ^{137}Cs/^{210}Pb dating.	1946 CE crustal earthquake<br>in central Vancouver Island (M7.3)	
	E1	—	1679–1703<br>(1σ range)	Seismite ages were estimated from the age model of Bringué et al. (2016)[11].<br>Enkin et al. (2013)[10] had created an age model based on ^{14}C ages from terrestrial plant fragments (leaves, cones, twigs, wood, and charcoal), the Mazama Ash, and the varve counting. Bringué et al. (2016)[11] developed this age model based on additional ^{14}C dates from plant fragments (leaves, twigs, and grass).	1700 CE Cascadia earthquake<br>Turbidite T1	Chang et al. (2003)[8]<br>Dallimore et al. (2005)[9]<br>Enkin et al. (2013)[10]<br>Bringué et al. (2016)[11]
	E2	—	1313–1343<br>(1σ range)			
	E3	—	1171–1227<br>(1σ range)		Turbidite T3	
Saanich Inlet	—	—	1946	Event age was estimated by varve counting, ^{137}Cs dating, and the position of the 1940 CE marker horizon (A Diatom, *Rhizosolenia* sp., first-appearance biohorizon).	1946 CE crustal earthquake<br>in central Vancouver Island (M7.3)	Blais-Stevens and Clague (2001)[12]
	—	—	1700	Event age was estimated by varve counting.<br>(Two deblis flow deposits collected at sites 6 km apart were dated to 1720 and 1725 CE.)	1700 Cascadia earthquake	
	Deblis-flow deposit (DFD)1	—	1575–1600	Event ages were estimated based on varve counting, radiocarbon dates, marker horizons (the Mazama volcanic ash and a Late Pleistocene outburst flood deposit) (Blais-Stevens et al., 2011)[13].		Blais-Stevens and Clague (2001)[12]<br>Blais-Stevens et al. (2011)[13]
	DFD2	—	1428–1517		Turbidate T2	
	DFD3	—	1123–1243			
	DFD4	—	1060–1136		Turbidate T3, buried soil T3	
Waatch / Nea Bay	SUB1	700±30	1260–1390*	Maximum age†: *In situ* plant remains including leaves and stems at the conatact between the subsidenced marsh peat and overlying tsunami sand layer.	1700 Cascadia earthquake	Peterson et al. (2013)[14]
Dicovery Bay	Bed 1	—	1585–1840	Maximum age: Section of *Alnus* sp. branch horizontal on lower bed contact<br>Minimum age: *Scirpus* cf. *acutus* rhizome<br>All ^{14}C ages and their stratigraphic relation to sand bed were presented by Willims et al.(2005)[15].	1700 Cascadia earthquake	Williams et al. (2005)[15]<br>Garrison-Laney (2017)[16]<br>Garrison-Laney and Miller (2017)[17]<br>All the event ages were calculated by Garrison-Laney (2017)[16].
	Bed 2	—	1320–1390	Maximum age: *Triglochin maritima* leaf bases below Bed 2<br>Minimum age: *T. maritima* leaf bases just above/within<br>All ^{14}C ages and their stratigraphic relation to sand beds were presented by Garrison-Laney, (2017)[16].		
	Bed 3	—	944–1280	Maximum age: Fragments of charcoal twig (?) above bed base(Willims et al., 2005)[15]; *Triglochin maritima*, twig, and *Bolboschoenus* sp. rhizome below Bed 4 (Garrison-Laney, 2017)[16].<br>Minimum age: Herbaceous root trough Bed 3 (Garrison-Laney, 2017)[16]; *Scirpus* cf. *acutus* rhizome (Williams et al., 2005)[15]	Buried soil W	
Lynch Cove	Layer A	—	1690–1830	Maximum age: *Triglochin maritima* leaf bases below Layer A<br>Minimum age: *T. maritima* leaf bases above/within Layer A	1700 Cascadia earthquake	Garrison-Laney (2017)[16]
	Layer B	—	1170–1230	Maximum age: Detrital twigs base/below Layer B<br>Minimum age: *Schoenoplectus* sp. rhizome within/above Layer B		
Southern Washington (Copalis River, Grays Harbor, Willapa Bay, Columbia RIver)	Buried soil Y	—	1700	Annual growth rings of trees (snags) killed by coseismic subsidence were dated. The time of tree death was estimated to be about 1700–1720 CE (Atwater et al., 1991[18]; Nelson et al., 1995[19]; Jacoby et al., 1997[21]; Yamaguchi et al., 1997[22]).		Atwater et al. (1991)[18]<br>Nelson et al. (1995)[19]<br>Atwater and Hemphill-Haley (1997)[20]<br>Jacoby et al. (1997)[21]<br>Yamaguchi et al. (1997)[22]<br>Atwater et al. (2004)[23]<br>Atwater and Griggs (2012)[24]
	Buried soil W	928±18	1030–1170*	Mean ages of three bark-bearing roots just below the top of the buried soil W (Atwater and Griggs, 2012)[24].		
Necanicum River	Tunami sand layer (TSL)1	480±60	1300–1630*	Maximum age†: Bulk peat below the TSL1 and 2a (Darienzo, 1991)[25]<br>Event name TSL are according to Peterson et al. (2010)[27].	1700 Cascadia earthquake	Darienzo (1991)[25]<br>Darienzo et al. (1994)[26]<br>Peterson et al. (2010)[27]<br>Peterson et al. (2013)[28]
	TSL2a	800±60	1040–1380*			
Cannon Beach (Ecola Creek)	1	90±40	1680–1806	Maximum age: Needles, moss, and seeds within sand layer 1<br>The upper bound of the age range is constrained by the year 1806 CE when written history began in coastal Oregon (Witter et al., 2008)[32].	1700 Cascadia earthquake	Peterson et al. (1993)[29]<br>Darienzo and Peterson (1995)[30]<br>Witter et al. (2008)[31]<br>Peterson et al. (2008)[32]
	2	570±40<br>790±50	1150–1430	Maximum age: Needles, moss, seeds, and twig within sand layer 2<br>The age range represents the sum of probabilities for two ages. The two ages do not overlap and meet criteria that define a statistical difference at the 95% level (Witter et al., 2008)[32].	Turbidates T2 or T3	
	3	990±60<br>1000±50<br>1050±40	970–1040	Maximum age: Conifer needles, needles, moss, and seeds within sand layer 3<br>The age range represents the pooled mean age of three statistically indistinguishable ages at the 95% level (Witter et al., 2008)[32].	Buried soil W, Turbidate T3	
Nehalem Bay	Contact A	128±9	1680–1930*	The weighted mean age of three tree stumps rooted in the peaty soil just below contact A<br>The stumps were inferred to have died by the coseismic subsidence (Nelson et al. 1995)[19].	1700 Cascadia earthquake	Nelson et al. (1995)[19]<br>Minor and Grant (1996)[33]<br>Nelson et al. (2020)[34]
	B	—	1008–1186	Maximum age: Abraded wood fragments and a *Picea sitchensis* needle just below contact B, Horizontal woody herb rhizome at contact B (Nelson et al., 2020)[34]<br>Minimum age: Growth position rhizomes of *Triglochin maritima* just above contact B<br>Event age was calculated from the maximum and minimum ages using the sequence model of OxCal (Nelson et al., 2020)[34].		
Netarts Bay	Ⅰ	270±60	After 1450*	Maximum age: Bulk peat just below the capping layer (Shennan et al., 1998)[37]	1700 Cascadia earthquake	Darienzo and Peterson (1990)[35]<br>Darienzo (1991)[25]<br>Darienzo et al. (1994)[26]<br>Long and Shennan (1998)[36]<br>Shennan et al. (1998)[37]
		350±60	1440–1650*	Maximum age†: Bulk peat (1MT) below the capping layer (Darienzo and Perterson, 1990)[37]		
		440±60	1390–1640*	Maximum age: Bulk peat just below the capping layer (Shennan et al., 1998)[37]		
	Ⅱ	660±60	1260–1410*	Maximum age†: Bulk peat (2MT) below the capping layer (Darienzo, 1991)[25]		
	Ⅲ	820±50	1040–1290*	Maximum age: Bulk peat just below the capping layer (Shennan et al., 1998)[37]		
Nestucca Bay	N1	180±40	After 1640*	Woody stem of *T. maritima* rooted within a few centimeters below the sand layer.<br>*T. maritima* was inferred to have died by the coseismic subsidence (Witter et al. 2009)[38].	1700 Cascadia earthquake	Darienzo et al. (1994)[26]<br>Witter et al. (2009)[38]
	N2	No data	—	Undated, insufficient evidence for great Cascadia earthquake (Witter et al., 2009)[38].		
	N3	980±40	990–1160*	Maximum age: Conifer needles deposited horizontally on the upper contact of peaty sediments buried by mud (Witter et al., 2009)[38].	Buried soil W, Turbidate T3	
Salmon River	Contact 1	157±17	Ater 1660*	The weighted mean of seven ages on *Juncus* cf. *balticus* in growth position at peat-mud contact 1.<br>The dated rush was inferred to have died by the coseismic subsidence (Nelson et al. 1995)[19].	1700 Cascadia earthquake	Nelson et al. (1995)[19]<br>Nelson et al. (2004)[39]
	2	1090±70	770–1150*	Maximum age: Peat just below peat-mud contact 2 (Nelson et al., 2004)[39]	Buried soil W?	
Siletz Bay	Sub/TSL 1	270±60	After 1450*	Maximum age†: Bulk peat (1MT) below the capping layer (Darienzo, 1991)[25]	1700 Cascadia earthquake	Darienzo (1991)[25]<br>Darienzo et al. (1994)[26]<br>Darienzo and Peterson (1995)[30]<br>Peterson et al. (2010)[40]
		480±60	1300–1630*	Maximum age†: Bulk peat (1MT) below the capping layer (Darienzo, 1991)[25]	1700 Cascadia earthquake	
Yaquina Bay	burial event 0'	160±60	After 1660*	Maximum age†: Bulk peat (0'MT) below the capping layer (Darienzo, 1991[25]; Darienzo et al., 1994[26])		Darienzo (1991)[25]<br>Darienzo et al. (1994)[26]<br>Darienzo and Peterson (1995)[30]<br>Graehl et al. (2015)[41]
	buried soil A	580±30	1300–1420*	Maximum age: Seeds just blow the buried soil-sand sheet conatact (Graehl et al., 2015)[41]		
Alsea Bay	Sand bed and peat-sand contact A	170±80<br>205±50	After 1640	Close maximum age: Herb flower bud just below the peat-sand contact A; Herb bud within peat below the peat-sand contact<br>Event age is calibrated from the mean of the two close maximum ages (Nelson et al., 2008)[44].	1700 Cascadia earthquake	Darienzo and Peterson (1995)[30]<br>Peterson and Darienzo (1996)[42]<br>Nelson et al. (2006)[43]<br>Nelson et al. (2008)[44]
	B	755±30<br>770±60<br>855±40	1100–1250	Close minimum age: *Picea stitchensis* leaves in fragments within the sand sheet; Decayed rhizome just above the sand sheet<br>Event age is calibrated from the mean of the two close maximum ages (Nelson et al., 2008)[44].<br>Close maximum age: Two separate *Juncus* sp. rhizomes at the peat-sand contact (Nelson et al., 2008)[44].		
Coos Bay (South Slough)	contact A	390±15	1445–1860	Maximum age: Fragments of soft woody herb stem just above the peat-mud conatact A<br>Event age was calculated using maximum limiting ages with OxCal (Milker et al., 2016)[49]	1700 Cascadia earthquake	Nelson (1992)[45]<br>Ota et al. (1995)[46]<br>Nelson and Personius (1996)[47]<br>Nelson et al. (1996)[48]<br>Milker et al. (2016)[49]
	B	560±20<br>1100±20	930–1405	Minimum age: Leaf sheaths, probably *Triglochin maritima*, just above the peat-mud conatact B<br>Maximum age: Abraded fragment of bark just above the peat-mud conatact B<br>Event age was calculated using maximum and minimum limiting ages with OxCal (Milker et al., 2016)[49]		
Coquille River	Buried soil 1	192±17	After 1660*	Maximum age: The weighted mean age of eight ages on *Carex* leaf bases just below the peat-mud contact 1 (Nelson, 1992[45]; Nelson et al. 1995[19]).	1700 Cascadia earthquake	Nelson (1992)[45]<br>Nelson et al. (1995)[19]<br>Witter et al. (2003)[50]
Bradley Lake	Disturbunce Event 1	—	1648–1800	Event age was calculated using sedimentation rates obtained from finely laminated mud facies.	1700 Cascadia earthquake	Kelsey et al. (2005)[51]
	DE2	—	950–1030	Event age was calculated using the V-sequence model of OxCal and time intervals between disturbance events estimated from sedimentation rates.		
Juan de Fuca Channel	T1	—	1590–1770			Goldfinger et al. (2012)[52]
	T2	—	1400–1560			
	T3	—	960–1180			
CSZ turbidite ages	OxCal combined age T1	—	1611–1750	Event ages were constrained with the Combine model of OxCal in 2σ ranges from ages of turbidites interpreted as correlative events.	1700 Cascadia earthquake	Goldfinger et al. (2012)[52]
	T2	—	1402–1502			
	T3	—	1110–1190		Buried soil W	
	Averaged age T1	—	1579–1811	Ages of turbidites interpreted as correlative events were averaged in 2σ ranges.	1700 Cascadia earthquake	
	T2	—	1377–1566			
	T3	—	1045–1271		Buried soil W	

*Ages calibrated using the radiocarbon calibration program OxCal 4.4[1, 2] with the IntCal20 radiocarbon calibration data set[3] at 2σ range and rounded to nearest 10 years.

†Only when there is no description about provenance interpretation of ^{14}C ages in previous studies, we regard ^{14}C ages from below and above the stratigraphic contacts (buried peat-mud, buried peat-sand) as maximum and minimum ages.

References for Supplementary Information

1. Bronk Ramsey, C. Bayesian analysis of radiocarbon dates. *Radiocarbon* **51**, 337–360 (2009).
2. Bronk Ramsey, C. Methods for Summarizing Radiocarbon Datasets. *Radiocarbon* **59**, 1809–1833 (2017).
3. Reimer, P. J. *et al.* The IntCal20 Northern Hemisphere Radiocarbon Age Calibration Curve (0–55 cal kBP). *Radiocarbon* **72**, 725–757 (2020).
4. Benson, B. E. et al. Tsunami Deposits beneath Tidal Marshes on Northwestern Vancouver Island, British Columbia. *Quat. Res.* **204**, 192–204 (1997).
5. Hutchinson, I. & Clague, J. Were they all giants? Perspectives on late Holocene plate-boundary earthquakes at the northern end of the Cascadia subduction zone. *Quat. Sci. Rev.* **169**, 29–49 (2017).
6. Clague, J. et al. Evidence for Late Holocene Tsunamis at Catala Lake, British Columbia. *J. Coast. Res.* **15**, 45–60 (1999).
7. Hutchinson, I., Guilbault J.-P., Clague, J. J. & Bobrowsky, P. T. Tsunamis and tectonic deformation at the northern Cascadia margin: a 3000-year record from Deserted Lake, Vancouver Island, British Columbia, Canada. *The Holocene* **10**, 429–439 (2000).
8. Chang, A. S., Patterson, R. T. & McNeely, R. Seasonal sediment and diatom record from Late Holocene laminated sediments, Effingham Inlet, British Columbia, Canada. *Palaios* **18**, 477–494 (2003).
9. Dallimore, A., Thomson, R. E. & Bertram, M. A. Modern to Late Holocene deposition in an anoxic fjord on the west coast of Canada: Implications for regional oceanography, climate and paleoseismic history. *Mar. Geol.* **219**, 47–69 (2005).

10. Enkin, R. J., Dallimore, A., Baker, J., Southon, J. R. & Ivanochko, T. A new high-resolution radiocarbon Bayesian age model of the Holocene and Late Pleistocene from core MD02-2494 and others, Effingham Inlet, British Columbia, Canada; with an application to the paleoseismic event chronology of the Cascadia Subduction Zone 1. Can. *J. Earth Sci.* **50**, 746–760 (2013).

11. Bringué, M. et al. High resolution dinoflagellate cyst record of environmental change in Effingham Inlet (British Columbia, Canada) over the last millennium. *Palaeogeogr. Palaeoclimatol. Palaeoecol.* **441**, 787–810 (2016).

12. Blais-Stevens, A. & Clague, J. J. Paleoseismic signature in late Holocene sediment cores from Saanich Inlet, British Columbia. *Mar. Geol.* **175**, 131–148 (2001).

13. Blais-Stevens, A., Rogers, G. C. & Clague, J. J. A revised earthquake chronology for the last 4,000 years inferred from varve-bounded debris-flow deposits beneath an inlet near Victoria, British Columbia. *Bull. Seismol. Soc. Am.* **101**, 1–12 (2011).

14. Peterson, C. D. *et al.* Coseismic subsidence and paleotsunami run-up records from latest Holocene deposits in the Waatch Valley, Neah Bay, northwest Washington, U.S.A.: Links to great earthquakes in the northern Cascadia margin. *J. Coast. Res.* **29**, 157–172 (2013).

15. Williams, H. et al. Multiple sources for late-Holocene tsunamis at Discovery Bay, Washington State, USA. *The Holocene* **15**, 60–73 (2005).

16. Garrison-Laney, C. Tsunamis and sea levels of the past millennium in Puget Sound. *PhD thesis. University of Washington* (2017).

17. Garrison-Laney, C. & Miller, I. Tsunamis in the Salish Sea: Recurrence, sources, hazards. *The Geological Society of America Field Guide.* **49**, 67–78 (2017).

18. Atwater, B. F., Stuivert, M. & Yamaguchi, D. K. Radiocarbon test of earthquake magnitude at the Cascadia subduction zone. *Nature* **353**, 156–158 (1991).

19. Nelson A. R. et al. Radiocarbon evidence for extensive plate-boundary rupture about 300 years ago at the Cascadia subduction zone. *Nature* **378**, 371–374 (1995).

20. Atwater, B. F. & Hemphill-Haley, E. Recurrence intervals for great earthquakes of the past 3,500 years at northeastern Willapa Bay, Washington. *U.S. Geological Survey Prof. Pap.* **1576** (1997).

21. Jacoby, G. C., Bunker, D. E. & Benson, B. E. Tree-ring evidence for an A.D. 1700 Cascadia earthquake in Washington and northern Oregon. *Geology* **25**, 999–1002 (1997).

22. Yamaguchi, D. K., Atwater, B. F., Bunker, D. E., Benson, B. E. & Reid, M. S. Tree-ring dating the1700 Cascadia earthquake. *Nature* **389**, 922–923 (1997).

23. Atwater, B. F. et al. Earthquake recurrence inferred from paleoseismology. *Dev. Quat. Sci.* **1**, 331–350 (2003).

24. Atwater, B. F. & Griggs, G. B. Deep-sea turbidites as guides to Holocene earthquake history at the Cascadia Subduction Zone—Alternative views for a seismic-hazard workshop. *US Geol. Surv. Open-File Rep.* **2012-1043** (2012).

25. Darienzo, M. E. Late Holocene Paleoseismicity along the Northern Oregon Coast. *PhD thesis. Portland State University* (1991).

26. Darienzo, M.E., Peterson, C.D. & Clough, C. Stratigraphic evidence for great subduction-zone earthquakes at four estuaries in northern Oregon. *J. Coast. Res.* **10**, 850–876 (1994).

27. Peterson, C. D., Jol, H. M., Horning, T. & Cruikshank, K. M. Paleotsunami inundation of a beach ridge plain: Cobble ridge overtopping and interridge valley flooding in seaside, Oregon, USA. *J. Geol. Res.* **2010**, 1–22 (2010).

28. Peterson, C. D., Clague, J. J., Carver, G. A. & Cruikshank, K. M. Recurrence intervals of major paleotsunamis as calibrated by historic tsunami deposits in three.

Nat. Hazards **68**, 321–336 (2013).

29. Peterson, C. D., Darienzo, M. E., Burns, S. F. & Burris, W. K. Field trip guide to Cascadia paleoseismic evidence along the northern Oregon coast: Evidence of subduction zone seismicity in the central Cascadia margin. *Oregon Geol.* **55**, 99–114 (1993).
30. Darienzo, M. E. & Peterson, C. D. Magnitude and frequency of subduction-zone earthquakes along the northern Oregon coast in the past 3,000 years. *Oregon Geol.* **57**, 3–12 (1995).
31. Witter, R.C. Prehistoric Cascadia tsunami inundation and runup at Cannon Beach, Oregon. *Oregon Department of Geology Mineral Industries Open-File Report* **O-08-12** (2008).
32. Peterson, C. D., Cruikshank, K. M., Jol, H. M. & Schlichting, R. B. Minimum runup heights of paleotsunami from evidence of sand ridge overtopping at Cannon Beach, Oregon, Central Cascadia Margin, U.S.A. *J. Sediment. Res.* **78**, 390–409 (2008).
33. Minor, R & Grant, W. C. Earthquake-induced subsidence and burial of late Holocene archaeological sites, northern Oregon coast. *American Antiquity*, **61**, 772–781 (1996).
34. Nelson, A. R. et al. Identifying the Greatest Earthquakes of the Past 2000 Years at the Nehalem River Estuary, Northern Oregon Coast, USA. *Open Quat.* **6**, 1–30 (2020).
35. Darienzo, M. E. & Peterson, C. D. Magnitude and frequency of subduction-zone earthquakes along the northern Oregon coast in the past 3,000 years. *Oregon Geol.* **57**, 3–12 (1995).
36. Long, A. J. & Shennan, I. Models of rapid relative sea-level change in Washington and Oregon, USA. *The Holocene* **8**, 129–142 (1998).

37. Shennan, I. *et al.* Tidal marsh stratigraphy, sea-level change and large earthquakes II—Submergence events during the last 3500 years at Netarts Bay, Oregon, USA. *Quat. Sci. Rev.* **17**, 365–393 (1998)

38. Witter, R. C., Hemphill-haley, E., Hart, R. & Gay, L. Tracking Prehistoric Cascadia Tsunami Deposits at Nestucca Bay, Oregon. *U.S. Geological Survey Award No. 08HQGR0076 Final Technical Rep.* (2009).

39. Nelson, A. R., Asquith, A. C. & Grant, W. C. Great earthquakes and tsunamis of the past 2000 years at the Salmon River estuary, central Oregon coast, USA. *Bull. Seismol. Soc. Am.* **94**, 1276–1292 (2004).

40. Peterson, C. D., Cruikshank, K. M., Schlichting, R. B. & Braunsten, S. Distal run-up records of latest Holocene paleotsunami inundation in alluvial flood plains: Neskowin and Beaver Creek, Oregon, central Cascadia margin, west coast U.S.A. *J. Coast. Res.* **26**, 622–634 (2010).

41. Graehl, N. A., Kelsey, H. M., Witter, R. C., Hemphill-haley, E. & Engelhart, S. E. Stratigraphic and microfossil evidence for a 4500-year history of Cascadia subduction zone earthquakes and tsunamis at Yaquina River estuary, Oregon, USA. *Geol. Soc. Am. Bull.* **127**, 211–226 (2015).

42. Peterson, C. D. & Darienzo, M. E. Discrimination of climatic, oceanic, and tectonic mechanisms of cyclic marsh burial, Alsea Bay, Oregon. *U.S. Geol. Surv. Prof. Pap.* **1560**, 115–146. (1996).

43. Nelson, a, Kelsey, H. & Witter, R. Great earthquakes of variable magnitude at the Cascadia subduction zone. *Quat. Res.* **65**, 354–365 (2006).

44. Nelson, A. R. *et al.* Great-earthquake paleogeodesy and tsunamis of the past 2000 years at Alsea Bay, central Oregon coast, USA. *Quat. Sci. Rev.* **27**, 747–768 (2008).

45. Nelson, A.R. Holocene tidal-marsh stratigraphy in south-central Oregon—Evidence

for localized sudden submergence in the Cascadia subduction zone. In: Fletcher, C. P., Wehmiller, J.F. (Eds.), Quaternary Coasts of the United States-Marine and Lacustrine Systems. Tulsa, Oklahoma. *Society for Sedimentary Geology Special Publication* **48**, 287–301 (1992).

46. Ota, Y., Umitsu, M., Kashima, K., Matsushima, Y. & Nelson, A. R. Interpreting an Earthquake History from the Stratigraphy of Late Holocene Intertidal Deposits in South Slough, Coos Bay, Oregon, USA. *J. Geography (Chigaku Zasshi)* **104**, 94–106 (1995).

47. Nelson, A. R. & Personius, S. F. Great-earthquake potential in Oregon and Washington—An overview of recent coastal geologic studies and their bearing on segmentation of Holocene ruptures, central Cascadia subduction zone. *U.S. Geol. Surv. Prof. Pap.* **1560**, 91–114. (1996).

48. Nelson, A. R., Shennan, I. & Long, A. J. Identifying coseismic subsidence in tidal-wetland stratigraphic sequences at the Cascadia subduction zone of western North America. *J. Geophys. Res.* **101**, 6115–6135 (1996).

49. Milker, Y. *et al.* Differences in coastal subsidence in southern Oregon (USA) during at least six prehistoric megathrust earthquakes. *Quat. Sci. Rev.* **142**, 143–163 (2016).

50. Witter, R. C., Kelsey, H. M. & Hemphill-Haley, E. Great Cascadia earthquakes and tsunamis of the past 6700 years, Coquille River estuary, southern coastal Oregon. *Geol. Soc. Am. Bull.* **115**, 1289–1306 (2003).

51. Kelsey, H. M., Nelson, A. R., Hemphill-Haley, E. & Witter, R. C. Tsunami history of an Oregon coastal lake reveals a 4600 yr record of great earthquakes on the Cascadia subduction zone. *Bull. Geol. Soc. Am.* **117**, 1009–1032 (2005).

52. Goldfinger, C. et al. *et al.* Turbidite Event History — Methods and Implications for Holocene Paleoseismicity of the Cascadia Subduction Zone. *U.S. Geol. Surv. Prof.*

Pap. **1661-F** (2012).
